# Mid to late‐life scores of depression in the cognitively healthy are associated with cognitive status and Alzheimer's disease pathology at death

**DOI:** 10.1002/gps.5470

**Published:** 2020-11-20

**Authors:** Andrew C. Robinson, Federico Roncaroli, Yvonne S. Davidson, James Minshull, Calvin Heal, Daniela Montaldi, Antony Payton, Michael A. Horan, Neil Pendleton, David M.A. Mann

**Affiliations:** ^1^ Division of Neuroscience & Experimental Psychology Faculty of Biology, Medicine and Health School of Biological Sciences The University of Manchester Salford Royal Hospital Salford UK; ^2^ Geoffrey Jefferson Brain Research Centre Manchester Academic Health Science Centre (MAHSC) Manchester UK; ^3^ Centre for Biostatistics Faculty of Biology, Medicine and Health School of Health Sciences The University of Manchester Manchester UK; ^4^ Division of Neuroscience & Experimental Psychology Faculty of Biology, Medicine and Health School of Biological Sciences The University of Manchester Manchester UK; ^5^ Division of Informatics, Imaging & Data Sciences Faculty of Biology, Medicine and Health School of Health Sciences The University of Manchester Manchester UK

**Keywords:** Alzheimer's disease, depression, early diagnosis, neuropathology, prodrome

## Abstract

**Objectives:**

Early diagnosis of Alzheimer's disease (AD) is essential for early interventions. Symptoms of depression could represent a prodromal stage of AD. Very early mood alterations may help to stratify those at highest risk of late‐life AD. We aim to investigate associations between baseline/longitudinal scores for depression, presence of cognitive impairment and/or AD pathology at death.

**Methods/Design:**

Between 1991 and 2015, participants from The University of Manchester Longitudinal Study of Cognition in Normal Healthy Old Age underwent 10 waves of assessment using the Geriatric Depression Scale (GDS). AD pathology at death was evaluated in 106 eligible cases. Analyses aimed to examine associations between GDS scores, cognitive status and AD pathology (as measured by Braak stage, Thal phase and CERAD).

**Results:**

Baseline GDS scores were significantly higher for those cognitively impaired at death than those cognitively normal. Significantly higher baseline GDS scores were found for those with greater Consortium to Establish a Registry for Alzheimer’s Disease (CERAD) scores than those with lower CERAD scores. Similarly, significantly higher baseline GDS scores were found for those with a greater Braak stage than those with lower tau burden. These correlations remained after controlling for age at death, education and *APOE* ε4, but were less robust. Mean longitudinal GDS scores associated with cognition but not pathology.

**Conclusions:**

GDS scores collected approximately 20 years before death were associated with cognitive status and AD pathology at death. We postulate that early AD‐related pathological change produces raised GDS scores due to an overlapping neural basis with depression, and that this may be considered as an early diagnostic marker for AD.

## 1 INTRODUCTION

Depression is a frequent co‐morbidity in patients with dementia and it presents unique challenges for clinicians who assess older, often cognitively impaired, adults.^1^ Large, population‐based studies have identified depression as a risk factor for dementia and, in particular, Alzheimer's disease (AD).^2^ Recent epidemiological studies have replicated this finding.^3^, ^4^, ^5^, ^6^ However, critically, few studies have had neuropathological confirmation of AD diagnosis.^7^

**Key points**

- Baseline depression scores correlate with cognition and Alzheimer pathology
- Mean longitudinal depression scores associate only with cognition at death
- Results suggest overlapping neural basis between Alzheimer's disease and depression

Depression affects about 50% of patients with AD,^8^ and the cognitive performance of patients with both AD and depression declines at a greater rate than of patients without depression.^9^ A history of depression has been shown to increase the risk of developing dementia.^10^ Early‐onset depression has been associated with an increased risk of AD.^11^, ^12^ However, late‐onset depression has been strongly implicated in AD, suggesting that depression is a prodrome of AD.^13^, ^14^, ^15^ One study highlighted that differences in depressive symptoms between those who do and those who do not go on to develop AD can be seen a decade before dementia diagnosis.^16^ However, it is noteworthy that other studies reported no depressive symptoms in the prodromal phase of AD.^17^

The major pathological changes associated with AD are abnormal accumulation of extracellular beta‐amyloid (Aβ), which is thought to be an initial event of AD, and secondary intracellular tau in the form of neurofibrillary tangles.^18^ It is thought that these pathological changes start many years before the onset of measurable cognitive decline.^19^ Both Aβ and tau have also been implicated in depression. Pre‐clinical AD is associated with low plasma levels of Aβ_42_ when compared with levels of Aβ_40_ and a low Aβ_42_:Aβ_40_ ratio is associated with depression in AD patients.^20^ This is exacerbated further in those individuals carrying the *APOE* ε4 allele.^21^ Levels of total tau and phosphorylated tau have been helpful in distinguishing mild cognitive impairment from major depressive disorder (MDD) which can be clinically difficult due to overlap of symptoms.^22^ In addition, a mutant R406W human tau mouse model exhibited changes in depression‐related behaviour which involved serotonergic neurons suggesting that tau may play a role in AD and depression.^23^ Conversely, a PET imaging study found no difference in amyloid binding between depressed and non‐depressed individuals,^24^ and other studies have also failed to associate amyloid burden with depression.^25^

Although clinical studies have significantly contributed to the understanding of the relationship between depression and AD, they lack the benefit of neuropathological confirmation of the disease. There are few studies with pathological confirmation of AD and they lack consensus in the established literature. A recent review concluded that cognitive dysfunction in MDD and AD share a common origin relating to the hippocampus and the prefrontal cortex; specifically in the breakdown of connectivity between these regions.^26^ It has previously been shown that AD patients with depression have a decreased number of neurons in the locus coeruleus compared to their non‐depressed counterparts.^27^ In addition, those with depression at intermediate stages of AD pathology (which is typically limbic) had a worse cognitive outcome than those without depression.^28^ Likewise, the presence of a past history of major depression seems to lead to an increase in Aβ plaques and tau tangles in AD patients when compared with AD patients without depression.^29^ Conversely, two large cohort studies that had brain donation as an end point, concluded that depression is not associated with any dementia‐related pathology.^30^, ^31^

The Yesavage Geriatric Depression Scale (GDS)^32^ is a widely used tool for assessing depressive symptoms in the elderly. Scores from the GDS have been shown to be reliable and relevant in those with AD.^33^, ^34^, ^35^ This study aims to establish whether GDS scores for depression, measured longitudinally and starting many years before death, associate with the degree of AD pathology found at death as measured by Braak stage,^36^ Consortium to Establish a Registry for Alzheimer’s Disease (CERAD) score,^37^ and Thal phase.^38^

## 2 MATERIALS AND METHODS

### 2.1 Participants and study design

Participants from The University of Manchester Longitudinal Study of Cognition in Normal Healthy Old Age (UMLCHA)^39^ were approached in 2003 for consent to brain donation. From the originally recruited total of 6542 healthy individuals (aged between 42 and 92 years), 312 individuals consented to brain donation. So far, 134 donations have been collected. Information pertaining to the clinical and pathological profile of this cohort has been previously described.^40^, ^41^

Between 1991 and 2003, participants underwent four face‐to‐face assessments of depression using the long form of the GDS which contains 30 YES/NO questions whose answers lead to a score which can be used to assess level of depressive symptoms. The cut off points are: 0–9 = normal; 10–19 = mild depression and 20–30 = severe depression. Between 2004 and 2015, participants underwent a further six telephone assessments of depressive symptoms using the short form of the GDS which contains 15 YES/NO questions. The cut off points are 0–5 = normal; 6–10 = suggestive of depression and 11–15 = severe. Both GDS30 and GDS15 have been previously validated as accurate means of assessing depression^32^ and scores on the GDS15 have been shown to correlate well with scores on GDS30.^42^

In addition, these participants (*n* = 100; Telephone Instrument for Cognitive Status [TICSm] data missing for six individuals) underwent five waves of cognitive assessment using the modified TICSm which contains 13 questions testing orientation, concentration, immediate and delayed memory, naming, calculation, comprehension and reasoning. The TICSm test^43^ has a maximum score of 39 and a score of 21 was used here as the cut‐off point, indicating cognitive impairment.^44^ Cognitive status at death was ascertained using a combination of the last TICSm score, patient notes obtained via participants' general practitioner and cause of death as recorded on the death certificate.

### 2.2 Pathological examination

Standard blocks of frontal, cingulate, temporal (including superior and middle temporal gyrus), parietal and occipital cortex, hippocampus, amygdala, corpus striatum, thalamus, midbrain, brainstem and cerebellum were cut from the fixed tissue and processed into wax blocks. One section was stained with haematoxylin‐eosin and further sections (6 µm) were immunostained for Aβ (Cambridge Bioscience, clone 4G8, 1:3000), tau proteins phosphorylated at Ser202 and Thr205 (P‐tau; Innogenetics, clone AT8, 1:750), phosphorylated α‐synuclein (#1175–Gift from Dr Masato Hasegawa at Tokyo Metropolitan Institute of Medical Science, Japan; 1:1000) and TAR DNA‐binding protein 43 (TDP‐43; Proteintech, 1:1000). For antigen retrieval, sections were either immersed in 70% formic acid for 20 min (for Aβ only) or microwaved in 0.1 M citrate buffer, pH 6.0 (all other antibodies) prior to incubation with primary antibody. Staining was visualised using the VECTASTAIN Elite ABC kit (Vector Laboratories) which is an avidin/biotin based amplification system. For Aβ and P‐tau, horse anti‐mouse biotinylated secondary antibody was used. For TDP‐43 and phosphorylated α‐synuclein, goat anti‐rabbit biotinylated secondary antibody was used.

Neuropathological assessment was performed by two experienced neuropathologists (Daniela Montaldi & Federico Roncaroli) who used consensus criteria to establish the presence and staging of common neurodegenerative diseases.^45^, ^46^, ^47^, ^48^, ^49^, ^50^, ^51^, ^52^, ^53^, ^54^, ^55^, ^56^, ^57^ In addition, vascular pathology was assessed using the Vascular Cognitive Impairment Neuropathology Guidelines.^58^ For the purpose of this study, we excluded all cases where the primary neuropathological diagnosis was not AD and also excluded AD cases where there was any secondary or concomitant pathology (other than cerebral amyloid angiopathy or small vessel disease). We included cases of Age‐Related Tau Astrogliopathy, Primary Ageing‐Related Tauopathy and Limbic‐predominant Age‐related TDP‐43 Encephalopathy owing to the fact that they are common ageing‐related pathologies. Of the 134 UMLCHA participants who had donated their brain, 106 were eligible. An overview of the pathology of eligible participants is available (Table S1).

### 2.3 Statistical analyses

The cohort was divided into pathology groups according to severity of AD pathology:

CERAD score: 0–A (low severity) versus B–C (high severity).

Thal phase: 0–3 (low severity) versus 4–5 (high severity).

Braak stage: 0–II (low severity) versus III–VI (high severity).

Differences between pathology groups in sex, cognitive impairment and presence of *APOE* ε4 allele(s) were analysed with the Chi‐squared test while those for age at death, and years of education were analysed with the T‐test.

Baseline GDS30 scores were not normally distributed. Therefore, differences between cognitive and pathology groups for baseline GDS30 scores were analysed with the Mann‐Whitney *U*‐Test. Further binary logistic regression analyses were used to determine whether the influence of sex, years of education and presence of *APOE* ε4 allele(s) affected the outcome.

To assess the association between longitudinal GDS scores and cognitive/pathology groups at death, we used binary logistic regression with mean depression score over the duration of the study as the independent variable of interest. For this analysis, both GDS15 and GDS30 measures were included, maximising the number of measures per patient. As these are on different scales, we standardised the scores by subtracting the mean and then dividing by the standard deviation of the sample. The mean was chosen as the measure of average due to the small number of depression scores over time per individual (mean number was 6). Covariates used in the model were sex, years of education and presence of *APOE* ε4 allele(s).

In all cases, a *p* value of <0.05 was considered significant.

## 3 RESULTS

### 3.1 Demographics

The demographics of the 106 eligible participants are shown in Table 1. There were no significant differences in sex, age at death or level of education between cognitive status groups, CERAD, Thal phase or Braak stage groups. As expected, a significantly greater proportion of cognitively impaired individuals were present in the high severity pathology groups for CERAD score (*χ* ^2^ = 27.2; *p* < 0.001), Thal phase (*χ* ^2^ = 6.1; *p* = 0.014) and Braak stage (*χ* ^2^ = 22.6; *p* < 0.001). Likewise, *APOE* ε4 allele(s) were more likely to be present in the high severity pathology groups for CERAD score (*χ* ^2^ = 5.7; *p* = 0.017), Thal phase (*χ* ^2^ = 10.1; *p* = 0.001) and Braak stage (*χ* ^2^ = 5.2; *p* = 0.023).

**TABLE 1:** Descriptive characteristics, stratified by AD neuropathological outcome and cognitive impairment at death, for eligible individuals from UMLCHA

	CI at death		CERAD score		Thal phase		Braak stage		Total cohort
	No	Yes	0–A	B–C	0–3	4–5	0–II	III–VI	
*N*	76	30	60	46	89	17	66	40	106
Female	54 (71.1%)	22 (73.3%)	40 (66.7%)	36 (78.3%)	64 (71.9%)	12 (70.6%)	47 (71.2%)	29 (72.5%)	76 (71.7%)
C.I at death	n/a	n/a	**5 (8.3%)**	**25 (54.3%)**	**21 (23.6%)**	**9 (52.9%)**	**8 (12.1%)**	**22 (55.0%)**	30 (28.3%)
Age at death (mean ± SD)	88.5 (±6.4)	89.7 (±4.1)	88.4 (±5.4)	89.4 (±6.3)	88.9 (±5.9)	88.5 (±5.7)	88.9 (±5.5)	88.8 (±6.4)	88.4 (±5.8)
Education in ISCED yrs (mean ± SD)	15.7 (±3.5)	15.6 (±3.8)	15.8 (±3.6)	15.4 (±3.6)	15.8 (±3.5)	14.8 (±4.1)	15.6 (±3.6)	15.7 (±3.5)	15.7 (±3.6)
One or more ε4 alleles present	18 (23.7%)	11 (36.7%)	**11 (18.3%)**	**18 (39.1%)**	**19 (21.3%)**	**10 (58.8%)**	**13 (19.7%)**	**16 (40.0%)**	29 (27.4%)

*Note*: Bold indicates significant difference between inclusive cognitive/pathology groups.

Abbreviations: AD, Alzheimer's disease; CI, cognitive impairment; CERAD, Consortium to Establish a Registry for Alzheimer’s Disease; UMLCHA, University of Manchester Longitudinal Study of Cognition in Normal Healthy Old Age.

### 3.2 Assessment of baseline GDS30 test scores and relationship to cognition and pathology at death.

Median baseline GDS30 score, mean age at baseline testing and mean number of years between test and death are shown in Table 2.

**TABLE 2:** Data pertaining to baseline GDS scores, age at testing and time between death and baseline GDS testing, stratified by cognitive impairment and AD neuropathological outcome

	CI at death		CERAD score		Thal phase		Braak stage		Total cohort
	No	Yes	0–A	B–C	0–3	4–5	0–II	III–VI	
Baseline									
Score (median/range)	**3.0 (25)**	**7.0 (13)**	**3.0 (25)**	**6.5 (20)**	4.0 (25)	5.0 (20)	**3.0 (25)**	**6.0 (20)**	4.0 (25)
Age at testing (mean ± SD)	68.4 (±5.5)	68.4 (±4.9)	68.2 (±5.2)	68.6 (±5.6)	68.0 (±5.3)	70.2 (±5.5)	68.1 (±5.2)	68.8 (±5.6)	68.4 (±5.3)
Years between test and death (mean ± SD)	20.5 (±4.0)	21.7 (±4.0)	20.8 (±3.8)	20.8 (±4.3)	**21.3 (±3.9)**	**18.7 (±4.0)**	21.2 (±3.7)	20.2 (±4.5)	20.8 (±4.0)

*Note*: Bold indicates significant difference between inclusive cognitive/pathology groups.

Abbreviations: AD, Alzheimer's disease; CI, cognitive impairment; CERAD, Consortium to Establish a Registry for Alzheimer’s Disease; GDS, Geriatric Depression Scale.

The mean age at baseline GDS30 test was 68.4 (±5.3) years which was 20.8 (±4.0) years before death.

**FIGURE 1:**
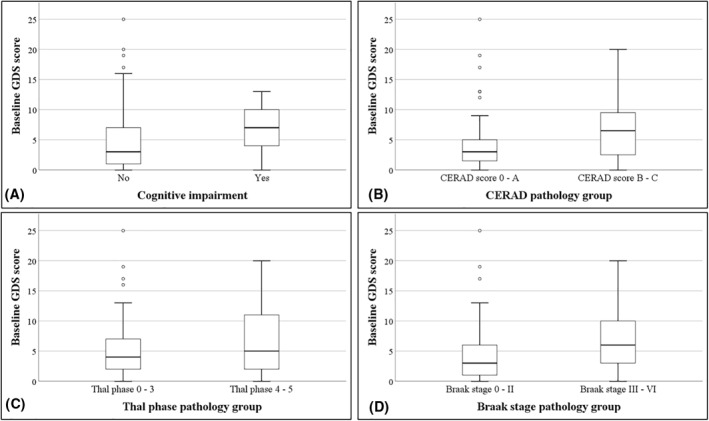
Boxplots comparing baseline Geriatric Depression Scale (GDS)30 scores between cognitive (Panel A) and Alzheimer's disease pathology groups (Panels B, C and D). The boxes represent the interquartile (IQ) range which contains the middle 50% of the records. The whiskers represent the highest and lowest values which are no greater than 1.5 times the IQ range. The line across the boxes indicates the median. Differences between cognitive and pathology groups for baseline GDS30 scores were analysed with the Mann–Whitney *U*‐Test

Those classified as having cognitive impairment at death scored higher on baseline GDS30 than their cognitively normal counterparts (*p* = 0.007). In addition, we found that baseline GDS30 scores were significantly higher in the severe AD pathology groups for CERAD and Braak stage when compared with the corresponding lower severity group (CERAD: *p* = 0.015; Braak stage; *p* = 0.018). However, this was not the case when examining Thal phase (*p* = 0.292; Figure 1).

Regression analyses showed that after accounting for sex, level of education and presence of *APOE* ε4 allele(s), the association between baseline GDS30 scores and cognitive impairment (*p* = 0.104), CERAD scores (*p* = 0.094) and Braak stage (*p* = 0.060) were no longer significant (Table 3).

**TABLE 3:** Logistic regression analysis assessing the impact of sex, level of education and presence of *APOE* ε4 allele(s) on the significant outcomes in baseline GDS scores between cognitive impairment, CERAD score and Braak stage groups

	Cognitive impairment at death			CERAD score			Braak stage		
	OR	95% CI	*p* value	OR	95% CI	*p* value	OR	95% CI	*p* value
Baseline GDS score	1.08	0.98–1.18	0.104	1.08	0.99–1.17	0.094	1.09	1.00–1.18	0.060
Sex	1.39	0.44–4.36	0.575	1.51	0.56–4.02	0.413	0.96	0.35–2.63	0.941
Education	1.00	0.87–1.14	0.946	0.95	0.85–1.07	0.429	0.97	0.86–1.09	0.599
Presence of *APOE* ε4	2.50	0.94–6.65	0.067	**2.98**	**1.17**–**7.60**	**0.023**	**3.16**	**1.24**–**8.05**	**0.016**

*Note*: Bold indicates significant difference.

Abbreviations: CI, confidence interval; CERAD, Consortium to Establish a Registry for Alzheimer’s Disease; GDS, Geriatric Depression Scale; OR, odds ratio.

### 3.3 Assessment of longitudinal GDS test scores and relationship to cognition and pathology at death.

Binary logistic regression analyses were conducted using cognitive or pathology groups as the outcome measure and mean score from the GDS30 and GDS15 time points as the independent variable of interest. Covariates used in the model were sex, years of education and presence of *APOE* ε4 allele(s).

The mean longitudinal GDS score was significantly higher in cognitively impaired individuals when compared with cognitively normal individuals (*p* = 0.047) at death. However, there were no differences in mean longitudinal GDS score between CERAD score (*p* = 0.185), Thal phase (*p* = 0.783) or Braak stage (*p* = 0.195) groups (Table 4).

**TABLE 4:** Logistic regression analysis assessing the impact of sex, level of education and presence of *APOE* ε4 allele(s) on the mean longitudinal GDS scores between cognitive impairment, CERAD score, Thal phase and Braak stage groups

Outcome	OR	95% CI	*p* value	AUROC (95% CI)
**Cognitive impairment**	**1.64**	**1.01**–**2.68**	**0.047**	**0.67 (0.56**–**0.78)**
CERAD score	1.06	0.97–1.16	0.185	0.67 (0.56–0.77)
Thal phase	1.09	0.58–2.08	0.783	0.71 (0.54–0.87)
Braak stage	1.36	0.85–2.18	0.195	0.67 (0.56–0.77)

*Note*: Bold indicates significant difference

Abbreviations: AUROC, area under ROC; CI, confidence interval; CERAD, Consortium to Establish a Registry for Alzheimer’s Disease; GDS, Geriatric Depression Scale; OR, odds ratio.

## 4 DISCUSSION

We have shown that scores from the baseline GDS30 test, undertaken approximately 20 years before death, associate with cognitive impairment and AD pathology at death. A general trend towards correlation remained after controlling for age at death, education level and presence of *APOE* ε4 allele(s). Although the difference in GDS30 score between the cognitive groups and also between the AD pathology groups is subtle and remains sub‐clinical for depression as measured by GDS, it nonetheless suggests that, if undertaken 20 years before death, the GDS is able to differentiate between, and could be predictive of, those who go on to develop AD pathology and associated cognitive impairment from those who will not.

Some studies have reported that cognitive test scores (usually of memory) are able to predict clinical dementia approximately 4–18 years before onset of symptoms.^59^, ^60^, ^61^, ^62^ Our findings show that a simple and widely used test for depression can also discriminate between those who become significantly cognitively impaired from those whose cognition remains relatively intact; and can do this many years before death.

A drawback to studies which are limited to clinical measures of dementia is the lack of neuropathological confirmation of disease type. Cognitive impairment is a ‘loose’ term encompassing many types of dementing illness. Here, instead, we ensured that our study group included only those with AD pathology and those with pathological findings that would be considered normal for the age of the subject. This gave us the opportunity to examine AD pathology alone as cases with possible concomitant pathology were excluded.

It is widely thought that the pathological processes underlying AD occur many years before any cognitive change that may lead to an individual seeking medical intervention.^63^ Many studies have shown that cognitive performance scores, obtained 1–6 years before death, can predict ultimate levels of AD pathology.^64^, ^65^, ^66^ However, most recently our group has shown that these findings can be extrapolated to approximately 20 years before death.^67^ The findings described in the present study, again, show that scores of cognitive performance, in this case GDS30 baseline scores, collected 20 years prior to death, associate with AD pathology at post‐mortem.

Neuroimaging and post‐mortem studies have attempted to elucidate the underlying pathophysiology of depression. Those with MDD show abnormal brain activity in frontal and temporal cortices, insula and cerebellum.^68^ In addition, those with MDD have moderate volume reduction in the hippocampus and striatum^69^ and a reduction of glia cell density in the prefrontal cortices and amygdalae.^70^, ^71^ However, the most striking findings are in the cingulate cortex where volume reduction has been shown early in MDD.^72^ Although the present study does not include individuals with known MDD, it is highly pertinent that the areas affected in MDD overlap somewhat with those affected relatively early on in AD by Aβ and tau; specifically the hippocampus, temporal cortex and amygdala. Moreover, the presence of depressive symptoms, not severe enough to be considered MDD, could result from neurofibrillary damage to serotonergic neurones in areas considered to be the first affected in AD, such as the dorsal raphe and locus coeruleus.^73^, ^74^, ^75^ It has previously been shown that damage to serotonergic neurones can lead to depressive symptoms in animal models.^23^ Thus, it would not be unreasonable to suggest that very early degenerative changes in these brain stem nuclei in humans could be responsible for generating mild depressive symptoms years before the onset of clinical AD. It is arguable therefore that the differences we report here in baseline GDS30 scores could reflect very early, subtle AD pathological changes, rather than the onset of a mild depressive disorder per se. This argument is bolstered by the fact that although the GDS30 baseline scores suggest mild depressive symptoms, they remain in the ‘normal’ range of the GDS protocol and do not indicate a diagnosis of depression.

Changes in neurogenesis could be another underlying mechanism explaining the very early differences in GDS scores between AD pathology groups. Both rodent models of AD and human AD post‐mortem tissue have shown decreased neurogenesis, especially within the ventral dentate gyrus of the hippocampus (and area of the brain implicated in both AD and MDD), while residual low‐level neurogenesis is likely to be still ongoing.^76^, ^77^ Interestingly, neurogenesis has also been implicated in the pathology of MDD and is argued to be one of the ways in which anti‐depressant medications exert their effect.^78^ One hypothesis could therefore be that early AD pathology induces localised disruption of neurogenesis in the dentate gyrus. This would prevent new cell proliferation and network integration, thereby causing early depressive symptoms and later, mild cognitive impairment, and finally, dementia.

It is worthy of note that only the baseline scores for GDS30 differentiated between CERAD and Braak stage pathology groups. The mean longitudinal scores did not show the same differences, possibly due to the deaths of participants during the study, which diminished the number of cases with available data, shrinking the study group and reducing the statistical power. Moreover, in the elderly, as age increases so does frequency of depression which likely confounds the longitudinal GDS scores. It is also very possible that those with less severe pathology still undergo the very early pathological changes associated with AD but at a later time point and fail to progress to the later stages of the disease owing to death. These individuals may therefore score higher on GDS at later time points rendering them more comparable to their severe pathology counterparts on the longitudinal GDS.

Sample size is a limitation imposed on the UMLCHA and the recruitment method (self‐selection) suggests that the study samples may not be representative of the general population. Similarly, the geographical areas covered by UMLCHA (Greater Manchester and Newcastle) may not reflect society as a whole. The diagnosis of cognitive impairment was not confirmed by a diagnostic clinical interview. However, a consensus on cognitive impairment at death was reached by experts using a wide range of in‐life measures. In addition, a lack of follow‐up closer to death means that cognitive decline may have been missed. Binary categorisation of the pathology outcomes was an unfortunate, but necessary, limitation owing to an insufficient sample size to consider them in their more natural, ordinal form. Strengths of the study include the choice of GDS as a tool to measure depression, owing to its reliability and relevance in AD,^33^, ^34^, ^35^ the longitudinal aspect of data collection and the ability to confirm diagnosis and assess neuropathological findings post‐mortem.

In conclusion, GDS30 scores collected approximately 20 years before death were associated with cognitive status at death and AD pathology, but mean longitudinal GDS scores were only associated with cognition and not with pathology. We postulate that the very early pathological changes associated with AD lead to higher scores on the GDS30 due to the overlap between brain regions implicated in depression and those compromised in very early AD. We suggest that a test battery combining assessments of cognition and symptoms of depression may well identify those individuals who will go on to develop AD, many years before they would have otherwise been diagnosed using the usual clinical assessments. However, for this to be robust, factors such as age at death, education level and *APOE* genotype would have to be considered.

## CONFLICTS OF INTEREST

The authors have declared that they have no conflict of interest to disclose.

## AUTHOR CONTRIBUTIONS

All authors have read the manuscript and have agreed to be listed as authors. Andrew C Robinson devised and designed the study, performed data/statistical analysis and wrote the paper. Federico Roncaroli finalised neuropathological diagnosis and contributed to writing the manuscript. James Minshull assisted with writing the manuscript and performed immunohistochemistry. Calvin Heal performed data/statistical analysis and assisted with preparation of the manuscript. Daniela Montaldi assisted with preparation of the manuscript and advised on neuropsychology. Yvonne S Davidson performed immunohistochemistry and assisted with preparation of the manuscript. Antony Payton assisted with preparation of the manuscript. Michael A Horan helped to finalise clinical cognitive impairment diagnosis and provided clinical data. David MA Mann finalised neuropathological diagnosis and contributed to writing the manuscript. Neil Pendleton finalised clinical cognitive impairment diagnosis and assisted with preparation of the manuscript.

## ETHICS COMMITTEE APPROVAL

The study was approved by Manchester Brain Bank Management Committee (REC reference **19/NE/0242**). Under conditions agreed with the Research Ethics Committee, The Manchester Brain Bank can supply tissue or data to researchers, without requirement for researchers to apply individually to the REC for approval.

## Supporting information

Supplementary Material 1

## Data Availability

The data that support the findings of this study are available from the corresponding author upon reasonable request.
