# Supplementary material for: Mid to late‐life scores of depression in the cognitively healthy are associated with cognitive status and Alzheimer's disease pathology at death
Source: Int J Geriatr Psychiatry. 2020 Nov 20;36(5):713–21. doi: 10.1002/gps.5470 (PMC8048934; doi:10.1002/gps.5470)
Supplement: Supplementary file 1 — Supplementary Material 1 [file GPS-36-713-s001.docx]

| **Case No.** | **CERAD score** | **Thal phase** | **Braak tau stage** | **Infarcts** | **CAA** | **Arterio** | **VCING** | **Braak LB stage** | **LATE-NC stage** | **PART** |
| --- | --- | --- | --- | --- | --- | --- | --- | --- | --- | --- |
| DPM04/12 | 0 | 0 | II | Absent | Absent | Present | Low | 0 | 0-1 | Definite |
| DPM05/05 | B | 3 | III | Absent | Absent | Absent | Low | 0 | 0-1 | Absent |
| DPM06/03 | A | 5 | IV | Absent | Absent | Absent | Low | 0 | 0-1 | Absent |
| DPM06/08 | B | 3 | IV | Absent | Absent | Absent | Low | 0 | 0-1 | Absent |
| DPM06/11 | C | 4 | IV | Absent | Absent | Absent | Low | 0 | 0-1 | Absent |
| DPM06/12 | A | 0 | I | Absent | Absent | Absent | Low | 3 (ageing-related only) | 0-1 | Possible |
| DPM06/18 | C | 4 | IV | Absent | Absent | Present | Low | 0 | 2-3 | Absent |
| DPM06/19 | C | 3 | IV | Absent | Present | Absent | Low | na | 0-1 | Absent |
| DPM07/07 | 0 | 0 | I | Absent | Present | Present | Moderate | 0 | 0-1 | Definite |
| DPM07/10 | C | 3 | III | Absent | Present | Present | Moderate | 0 | 0-1 | Absent |
| DPM07/13 | 0 | 0 | 0 | Absent | Absent | Present | Low | 0 | 0-1 | Absent |
| DPM08/04 | A | 4 | I | Absent | Absent | Absent | Low | 0 | 0-1 | Absent |
| DPM08/28 | A | 3 | 0 | Present | Absent | Present | High | 0 | 0-1 | Absent |
| DPM08/29 | A | 5 | II | Absent | Present | Present | Moderate | 0 | 0-1 | Absent |
| DPM09/05 | 0 | 0 | II | Absent | Absent | Absent | Low | 0 | 0-1 | Definite |
| DPM09/07 | B | 4 | III | Absent | Absent | Present | Low | 0 | 2-3 | Absent |
| DPM09/15 | C | 4 | IV | Absent | Absent | Absent | Low | 0 | 0-1 | Absent |
| DPM09/21 | B | 3 | II | Absent | Present | Present | Moderate | 0 | 0-1 | Absent |
| DPM09/22 | B | 3 | III | Absent | Present | Absent | Low | 0 | 0-1 | Absent |
| DPM09/24 | 0 | 0 | 0 | Absent | Absent | Absent | Low | 0 | 0-1 | Absent |
| DPM09/26 | 0 | 0 | I | Absent | Absent | Absent | Low | 0 | 0-1 | Definite |
| DPM09/31 | 0 | 0 | I | Absent | Absent | Absent | Low | 0 | 0-1 | Definite |
| DPM10/07 | B | 5 | IV | Present | Present | Present | High | 0 | 2-3 | Absent |
| DPM10/08 | B | 5 | III | Absent | Present | Present | Moderate | 0 | 2-3 | Absent |
| DPM10/16 | B | 3 | III | Present | Absent | Present | High | 0 | 0-1 | Absent |
| DPM10/40 | B | 3 | III | Absent | Absent | Absent | Low | 0 | 0-1 | Absent |
| DPM11/06 | A | 1 | II | Absent | Absent | Absent | Low | 0 | 0-1 | Possible |
| DPM11/07 | 0 | 0 | 0 | Absent | Absent | Absent | Low | 0 | 0-1 | Absent |
| DPM11/15 | 0 | 0 | I | Absent | Absent | Present | Low | 0 | 0-1 | Definite |
| DPM11/20 | B | 1 | II | Absent | Absent | Absent | Low | 0 | 0-1 | Possible |
| DPM11/22 | 0 | 0 | 0 | Absent | Absent | Present | Low | 0 | 0-1 | Absent |
| DPM11/25 | 0 | 0 | II | Absent | Absent | Absent | Low | 0 | 0-1 | Definite |
| DPM11/27 | A | 1 | II | Absent | Absent | Absent | Low | 0 | 0-1 | Possible |
| DPM11/29 | 0 | 0 | II | Absent | Absent | Absent | Low | 0 | 0-1 | Definite |
| DPM12/09 | A | 1 | I | Absent | Absent | Absent | Low | 0 | 0-1 | Possible |
| DPM12/23 | 0 | 0 | I | Absent | Absent | Absent | Low | 0 | 0-1 | Definite |
| DPM12/28 | A | 3 | II | Absent | Present | Absent | Low | 0 | 0-1 | Absent |
| DPM12/33 | B | 4 | III | Absent | Present | Present | Moderate | 0 | 0-1 | Absent |
| DPM12/34 | B | 1 | III | Absent | Absent | Absent | Low | 0 | 0-1 | Possible |
| DPM12/35 | A | 3 | II | Absent | Present | Absent | Low | 0 | 0-1 | Absent |
| DPM13/10 | B | 3 | V | Absent | Absent | Absent | Low | 0 | 0-1 | Absent |
| DPM13/11 | B | 3 | IV | Absent | Present | Absent | Low | 0 | 2-3 | Absent |
| DPM13/12 | A | 1 | II | Absent | Absent | Absent | Low | 0 | 0-1 | Possible |
| DPM13/16 | B | 5 | III | Absent | Present | Absent | Low | 0 | 2-3 | Absent |
| DPM13/17 | B | 3 | III | Present | Present | Present | High | 0 | 2-3 | Absent |
| DPM13/21 | 0 | 0 | II | Absent | Absent | Present | Low | 0 | 0-1 | Definite |
| DPM13/22 | 0 | 0 | II | Present | Present | Absent | High | 0 | 0-1 | Definite |
| DPM13/23 | A | 0 | II | Absent | Absent | Absent | Low | 0 | 0-1 | Definite |
| DPM13/31 | 0 | 0 | II | Absent | Absent | Absent | Low | 0 | 0-1 | Definite |
| DPM13/32 | A | 1 | 0 | Absent | Absent | Present | Low | 0 | 0-1 | Absent |
| DPM13/35 | 0 | 0 | I | Present | Absent | Absent | Moderate | 0 | 0-1 | Definite |
| DPM13/36 | A | 3 | II | Absent | Present | Absent | Low | 0 | 0-1 | Absent |
| DPM14/01 | B | 4 | IV | Absent | Absent | Absent | Low | 0 | 0-1 | Absent |
| DPM14/04 | A | 1 | 0 | Absent | Absent | Absent | Low | 0 | 0-1 | Absent |
| DPM14/06 | B | 1 | II | Absent | Present | Absent | Low | 0 | 0-1 | Possible |
| DPM14/11 | 0 | 0 | I | Absent | Absent | Absent | Low | 0 | 0-1 | Definite |
| DPM14/14 | B | 3 | II | Absent | Present | Absent | Low | 0 | 0-1 | Absent |
| DPM14/16 | A | 1 | 0 | Absent | Absent | Absent | Low | 0 | 0-1 | Absent |
| DPM14/20 | 0 | 0 | 0 | Absent | Absent | Absent | Low | 0 | 0-1 | Absent |
| DPM14/29 | B | 1 | II | Absent | Absent | Absent | Low | 0 | 0-1 | Possible |
| DPM14/42 | B | 3 | III | Absent | Absent | Absent | Low | 0 | 2-3 | Absent |
| DPM14/46 | 0 | 0 | 0 | Absent | Absent | Absent | Low | 0 | 0-1 | Absent |
| DPM15/01 | A | 1 | I | Absent | Absent | Present | Low | 0 | 0-1 | Possible |
| DPM15/05 | A | 1 | I | Absent | Present | Absent | Low | 0 | 0-1 | Possible |
| DPM15/11 | C | 5 | VI | Absent | Present | Present | Moderate | 0 | 2-3 | Absent |
| DPM15/14 | B | 3 | III | Absent | Present | Present | Moderate | 0 | 0-1 | Absent |
| DPM15/15 | 0 | 0 | II | Present | Absent | Absent | Moderate | 0 | 0-1 | Definite |
| DPM15/19 | B | 2 | III | Present | Absent | Present | High | 0 | 0-1 | Possible |
| DPM15/26 | A | 3 | 0 | Absent | Absent | Absent | Low | 0 | 0-1 | Absent |
| DPM15/28 | 0 | 0 | II | Present | Absent | Absent | Moderate | 0 | 0-1 | Definite |
| DPM15/30 | A | 2 | II | Absent | Absent | Absent | Low | 0 | 0-1 | Possible |
| DPM15/31 | A | 3 | I | Absent | Absent | Absent | Low | 0 | 0-1 | Absent |
| DPM15/42 | B | 5 | III | Absent | Present | Absent | Low | 0 | 0-1 | Absent |
| DPM15/44 | A | 1 | I | Absent | Absent | Absent | Low | 0 | 2-3 | Possible |
| DPM16/01 | B | 4 | III | Absent | Absent | Absent | Low | 0 | 2-3 | Absent |
| DPM16/03 | C | 3 | V | Absent | Present | Absent | Low | 0 | 0-1 | Absent |
| DPM16/12 | 0 | 0 | II | Absent | Absent | Absent | Low | 0 | 0-1 | Definite |
| DPM16/13 | C | 5 | V | Absent | Absent | Absent | Low | 0 | 0-1 | Absent |
| DPM16/15 | B | 2 | II | Present | Present | Present | High | 0 | 0-1 | Possible |
| DPM16/18 | B | 3 | I | Absent | Absent | Present | Low | 0 | 0-1 | Absent |
| DPM16/19 | B | 2 | II | Present | Absent | Present | High | 0 | 0-1 | Possible |
| DPM16/23 | 0 | 0 | I | Absent | Absent | Absent | Low | 0 | 0-1 | Definite |
| DPM16/24 | 0 | 0 | I | Absent | Absent | Absent | Low | 0 | 0-1 | Definite |
| DPM16/30 | A | 3 | II | Absent | Absent | Absent | Low | 0 | 0-1 | Absent |
| DPM16/37 | B | 2 | III | Absent | Present | Absent | Low | 0 | 2-3 | Possible |
| DPM16/41 | A | 2 | I | Absent | Present | Absent | Low | 0 | 0-1 | Possible |
| DPM16/45 | B | 5 | III | Absent | Present | Absent | Low | 0 | 2-3 | Absent |
| DPM17/04 | 0 | 0 | I | Absent | Absent | Absent | Low | 0 | 0-1 | Definite |
| DPM17/09 | 0 | 0 | I | Absent | Absent | Absent | Low | 0 | 0-1 | Definite |
| DPM17/22 | A | 2 | II | Absent | Absent | Absent | Low | 0 | 0-1 | Possible |
| DPM17/29 | A | 2 | II | Absent | Absent | Absent | Low | 0 | 0-1 | Possible |
| DPM17/32 | B | 3 | IV | Absent | Absent | Absent | Low | 0 | 0-1 | Absent |
| DPM17/34 | 0 | 0 | I | Absent | Absent | Absent | Low | 3 (ageing-related only) | 0-1 | Definite |
| DPM17/38 | A | 1 | II | Absent | Absent | Absent | Low | 0 | 0-1 | Possible |
| DPM18/03 | 0 | 0 | 0 | Absent | Absent | Absent | Low | 0 | 0-1 | Absent |
| DPM18/09 | B | 3 | IV | Absent | Present | Present | Moderate | 0 | 0-1 | Absent |
| DPM18/11 | A | 1 | I | Absent | Absent | Absent | Low | 0 | 0-1 | Possible |
| DPM18/18 | A | 2 | III | Absent | Absent | Absent | Low | 0 | 0-1 | Possible |
| DPM18/32 | B | 2 | II | Absent | Absent | Present | Low | 0 | 0-1 | Possible |
| DPM18/38 | A | 3 | IV | Present | Present | Present | High | 0 | 0-1 | Absent |
| DPM19/09 | 0 | 1 | II | Absent | Absent | Present | Low | 0 | 0-1 | Absent |
| DPM19/12 | C | 3 | VI | Absent | Absent | Absent | Low | 0 | 2-3 | Absent |
| DPM19/20 | C | 3 | VI | Absent | Absent | Present | Low | 0 | 0-1 | Absent |
| DPM19/21 | B | 3 | III | Absent | Absent | Present | Low | 0 | 0-1 | Absent |
| DPM19/25 | B | 3 | VI | Absent | Present | Present | Moderate | 0 | 0-1 | Absent |
| DPM19/26 | B | 2 | III | Present | Absent | Absent | Moderate | 0 | 0-1 | Possible |

**Supplementary table 1** – Neuropathological overview of the 106 eligible participants (**CAA** – moderate/severe occipital leptomeningeal cerebral amyloid angiopathy; **Arterio** - moderate/severe arteriolosclerosis in occipital white matter; **VCING** - Vascular cognitive impairment neuropathology guidelines outcome; **Braak LB stage** – Braak Lewy body stage; **LATE-NC stage** – Limbic-predominant age-related TDP-43 encephalopathy – neuropathological change stage; **PART** – Primary age-related tauopathy)
